# An efficient method for transgenic callus induction from *Vitis amurensis* petiole

**DOI:** 10.1371/journal.pone.0179730

**Published:** 2017-06-22

**Authors:** Tingting Zhao, Zemin Wang, Lingye Su, Xiaoming Sun, Jun Cheng, Langlang Zhang, Sospeter Karanja Karungo, Yuepeng Han, Shaohua Li, Haiping Xin

**Affiliations:** 1 Key Laboratory of Plant Germplasm Enhancement and Specialty Agriculture, Wuhan Botanical Garden, Chinese Academy of Sciences, Wuhan, P.R. China; 2 University of Chinese Academy of Sciences, Beijing, P.R. China; 3 Beijing Key Laboratory of Grape Sciences and Enology, CAS Key Laboratory of Plant Resources, Institute of Botany, Chinese Academy of Sciences, Beijing, P.R. China; Key Laboratory of Horticultural Plant Biology (MOE), CHINA

## Abstract

Transformation is the main platform for genetic improvement and gene function studies in plants. However, the established somatic embryo transformation system for grapevines is time-consuming and has low efficiency, which limits its utilization in functional genomics research. *Vitis amurensis* is a wild *Vitis* species with remarkable cold tolerance. The lack of an efficient genetic transformation system for it has significantly hindered the functional identification of cold stress related genes in the species. Herein, an efficient method was established to produce transformed calli of *V*. *amurensis*. Segments of petioles from micropropagated plantlets of *V*. *amurensis* exhibited better capacity to differentiate calli than leaf-discs and stem segments, and thus was chosen as target tissue for *Agrobacterium*-mediated transformation. Both *neomycin phosphotransferase II* (*NPTII*) and *enhanced green fluorescent protein* (*eGFP*) genes were used for simultaneous selection of transgenic calli based on kanamycin resistance and eGFP fluorescence. Several parameters affecting the transformation efficiency were optimized including the concentration of kanamycin, *Agrobacterium* stains, bacterial densities, infection treatments and co-cultivation time. The transgenic callus lines were verified by checking the integration of *NPTII* gene into calli genomes, the expression of *eGFP* gene and the fluorescence of eGFP. Up to 20% of the petiole segments produced transformed calli after 2 months of cultivation. This efficient transformation system will facilitate the functional analysis of agronomic characteristics and related genes not only in *V*. *amurensis* but also in other grapevine species.

## Introduction

Genetic transformation systems are one of the most important platforms that are used for genetic improvement and also for functional analyses of genes in plants. As one of the most important fruit crops cultivated worldwide, the regeneration and genetic transformation of grapevines has been widely studied. Somatic embryogenesis has been reported in several *Vitis* species and derived from various explants such as ovules [1, 2], anthers [2], leaves and petioles [3–5], and tendrils [6]. Organogenesis was reported using leaves and petioles as explants [5, 7, 8, 9]. *Agrobacterium*-mediated genetic transformation of grape has been reported, and the factors affecting transformation efficiency such as genotype, *Agrobacterium* strain and selection regime have been studied [10–19]. Genotype significantly affected regeneration and transformation [14, 20], and suitable strategies need to be developed for specific *Vitis* species.

To date, anther-derived somatic embryogenesis has been the primary method used for transformation of *V*. *vinifera* cultivars such as ‘Thompson Seedless’ [18, 21], but the time required and low efficiency of this procedure has limited its usefulness in functional genomics studies in grapevines. A few studies have used transformation systems to illustrate gene function in grapevines. Transgenic ‘Chardonnay’ containing magainin genes exhibited significant reductions in crown gall symptoms compared to non-transformed controls [22]. The expression of stilbene synthase gene from *V*. *pseudoreticulata* increased the resveratrol concentration in transgenic ‘Thompson Seedless’ [23]. The function of *VvCBF4* in cold tolerance was verified and its possible downstream genes were identified in transgenic *V*. *vinifera* cv. Freedom [24]. Zhou et al. reported that overexpressing of *VpPUB23* in ‘Thompson Seedless’ decreased resistance to powdery mildew[18].

*V*. *amurensis* is a wild *Vitis spp*. species with remarkable cold tolerance [25]. Numerous candidate genes that correlated to low temperature responses in *V*. *amurensis* were identified [26, 27], but the lack of a suitable transformation system hindered the functional studies of key genes for cold hardiness in the species. Some cold stress-related transcription factors were transformed into *Arabidopsis* to identify their functions and related signal pathways [28–32]. However, due to the different genomic backgrounds between *Arabidopsis* and *V*. *amurensis*, it was difficult to illustrate specific signal transduction pathways under cold stress in *V*. *amurensis* by using the *Arabidopsis* transformation system.

In the present study, a quick and efficient system was developed to produce transformed calli in *V*. *amurensis*. Micropropagated plantlets of *V*. *amurensis* (collected from Changbai Mountain in Jilin province, Northeastern China) were used as parent material. A vector, pSAK277-eGFP, containing the selective marker gene *NPTII* (conferring resistance to kanamycin) and reporter gene *eGFP* were applied during *Agrobacterium*-mediated transformation. Suitable explants were chosen by comparing the callus derived from leaf-discs, stem segments and petiole segments. Several factors that influenced transgenic efficiency were evaluated including *Agrobacterium* strains, selection regime, infection and co-cultivation conditions. Positive calli were confirmed by PCR analysis and eGFP fluorescence observation. The possible use of this transformation system is also discussed.

## Materials and methods

### Plant material and growth conditions

Micropropagated plantlets of *V*. *amurensis*, and of *V*. *vinifera* cv. Muscat Hamburg and Centennial Seedless, were grown on 1/2 MS [33] solid medium supplemented with 30 g L^−1^ sucrose, 7 g L^−1^ agar and 0.2 mg L^−1^ IAA in conical flasks (35 mL) in a growth chamber at 26°C. The average photosynthetic photon flux was 100 μmol m^−2^ s^−1^ with a 16 h light and 8 h dark cycle. Five weeks old plantlets were used for explant collection. The non-transformed and transformed explants were maintained on 90×15 mm Petri dish with 25 mL modified B5 solid medium supplemented with 0.25 g L^−1^ casein hydrolysate, 5 g L^−1^ PVP, 0.58 g L^−1^ MES (pH 5.8), 30 g L^−1^ sucrose, 0.1 mg L^−1^ 6-BA, 0.1 mg L^−1^ 2, 4-D, 7 g L^−1^ agar and 0–20 mg L^−1^ kanamycin. The explants were cultured in the dark at 26°C.

### Selection of suitable explants and kanamycin concentration for transformation

The leaf-discs (5 mm in diameter), and stem and petiole segments (3–5 mm in length) of *V*. *amurensis* were used for evaluating its differentiation capacity. Explants were grown under the above-mentioned conditions without kanamycin. A total of 30 leaf-discs, stem segments and petiole segments were cultivated in each Petri dish, respectively. Each experiment was repeated three times. The differentiation rate and status of calli were recorded after 30 days of cultivation.

To establish an appropriate kanamycin concentration for selecting transformed calli, petiole segments were cultivated in medium with different concentrations of kanamycin (0, 5, 10, 15 and 20 mg L^-1^). A total of 30 petiole segments were cultivated in each Petri dish at each kanamycin concentration with three replicates of each dish. The differentiation rate of petiole segments was evaluated after 30 days of cultivation. The optimal concentration of kanamycin (20 mg L^-1^) that inhibited the development of callus was selected and used in all subsequent transformation experiments to select transformed callus.

### Vector construction

The *eGFP* gene was amplified from pEZS-NL vector using the primers eGFP-F1 (5'-CGGAATTCATGGTGAGCAAGGGCGAG-3') and eGFP-R1 (5'-CCGCTCGAGCTTG TACAGCTCGTCCATGCC-3'), resulting in an eGFP DNA fragment without a stop codon. After double digestion by EcoRI and XhoI, the fragment was ligated into pSAK277 vector downstream of the cauliflower mosaic virus (CaMV) 35S promoter and upstream of FLAG tags (with stop codon, S1 Fig). The *NPTII* gene in pSAK277 vector was used as a selective marker for transgenic calli during petiole segment transformation.

### Transformation and generation of transgenic callus from petiole segments

The plasmid of pSAK277-eGFP was transformed into *A*. *tumefaciens* strain EHA105 by electroporation. *Agrobacterium* was then cultured overnight in YEP liquid medium (10 g L^-1^ tryptone, 5 g L^-1^ NaCl, 10 g L^-1^ yeast extract) at 28°C on a rotary shaker (220 rpm) supplemented with 50 mg L^-1^ rifampicin and 100 mg L^-1^ spectinomycin. Prior to transformation, *Agrobacterium* were centrifuged at 5,000 rpm for 10 min and washed twice with sterile, distilled water and then re-suspended in 50 mL liquid MS medium containing 100 μM AS (acetosyringone). The suspended cells were incubated on a shaker (120 rpm) at 28°C for 3 hours. The suspended cells (OD_600_ = 0.5) were then used for transformation. The petiole segments (3–5 mm) were submerged in bacterial solution for 8 min and then blotted dry on filter paper. The petiole segments were subsequently placed on modified B5 solid medium and co-cultivated 2 days at 26°C in the dark. The fragments were then washed twice in sterile, distilled water containing 400 mg L^-1^ carbenicillin and 400 mg L^-1^ cefotaxime. The washed petiole segments were blotted dry on filter paper and then transferred to Petri dishes containing 26 mL modified B5 solid medium (400 mg L^−1^ carbenicillin, 400 mg L^−1^ cefotaxime and 20 mg L^−1^ kanamycin) and cultured at 26°C in the dark. The petioles segments with kanamycin-resistant calli or only calli were transferred monthly into fresh modified B5 solid medium with 200 mg L^−1^ carbenicillin and 200 mg L^−1^ cefotaxime.

Another set of *A*. *tumefaciens* strains, LBA4404 and GV3101, also transformed with pSAK277-eGFP, were used for petiole segment transformation to evaluate transformation efficiency. The EHA105 strain containing pSAK277-eGFP plasmid was used to optimize the parameters such as the concentration of the bacteria cells (OD_600_ = 0.5, 1.0, 1.5 and 2.0), the infection time (4, 8, 12 and 16 min) and the co-cultivation time (1, 2, 3 and 4 days), respectively, during petiole segment transformation. Thirty petiole segments were cultured per Petri dish and three replicates dishes were established for each transformation condition. After 6 weeks, the kanamycin-resistant calli with observable eGFP fluorescence were counted as positive calli. The transformation frequency was defined as the percentage of petiole segments that produced the transgenic callus.

### eGFP fluorescence observation

The eGFP fluorescence in kanamycin-resistant calli was monitored using a fluorescence microscope (ECLIPSE 80i, Nikon). GFPuv was excited at 465–495 nm and emitted through a 515–555 nm bandpass filter. The callus that grew well on kanamycin containing medium and with detectable eGFP fluorescence was designated as a positive callus. The possible chimera of positive calli was detected. Positive calli were incubated in enzyme solution (2% Cellulase R10, 0.5% Macerozyme R10, 0.4M Mannitol, 20mM KCl, 10mM CaCl_2_, 0.1% BSA, 20mM MES, pH 5.8) at 26°C in dark for 7 hours [34, 35]. The percentage of cells with eGFP fluorescence was counted from more than 100 cells. The experiment was replicated three times and a total of five callus lines (derived from different petiole segments) were checked.

### Molecular analysis of transgenic callus lines

About 0.5 g calli from five positive and one non-transformed callus lines was collected, respectively. The total genomic DNA was isolated from these samples by using Plant Genomic DNA kit (Cat: DP305-03, TIANGEN, China). PCR was carried out to detect the insertion of *NPTII* gene in the genomes of calli using the forward primer NPTII-F (5'-ATTACCTTATCCGCAACTTCTTTACC-3') and a reverse primer NPTII-R (5'-AGCCCCTGATGCTCTTCGTC-3').

Total RNA was extracted from collected samples with Column Plant RNAout 2.0 (Cat: 90404–50, TIANDZ, China). First cDNA strands were synthetized by TransScript^®^ One-step gDNA Remover and cDNA synthesis SuperMix (Transgen, China). A pair of primers were designed according to the eGFP DNA sequences (eGFP-F2: 5'-ATGGTGAGCAAGGGGA G -3') and (eGFP-R2: 5'-CTTGTACAGCTCGTCCATGCC-3') to validate the expression of *eGFP* gene in transgenic calli.

The PCR was performed the following conditions: 94°C for 3 min; 35 cycles at 94°C for 30 s, 55°C for 30 s and 72°C for 1 min; and 72°C for 10 min. The plasmid DNA of pSAK277-eGFP was used as a positive control while the DNA or cDNAs from non-transformed calli were used as a negative control. Amplified fragments were detected by UV after electrophoresis on agarose gel (1.1%, w/v) with Ethidium Bromide.

### Statistical analysis

The reported experimental data represent at least three independent biological repeats. Where appropriate, results are reported as mean values ± standard errors (SE). Mean comparisons were calculated by Student’s t-test, and *P*-values and data sizes were indicated in the figure legends.

## Results

### Selection of suitable types of explants for transformation

**Fig 1:**
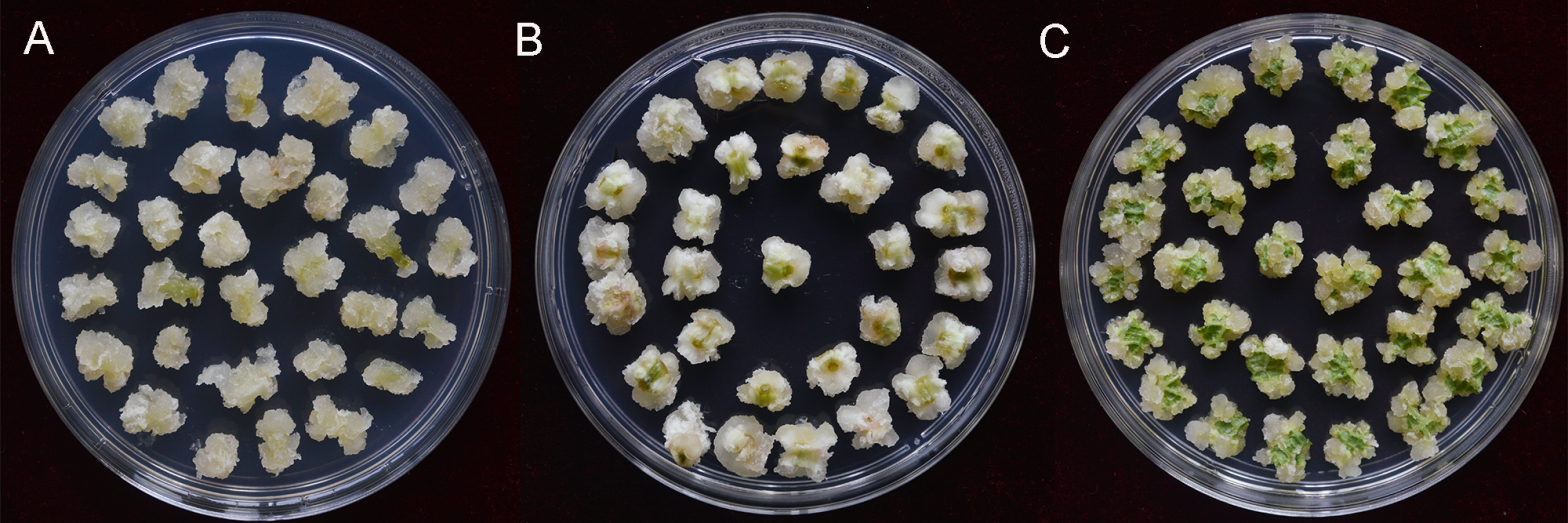
Selection of optimal explant types for transformation. Calli generated from: A: petiole segments; B: stem segments; C: leaf discs.

Three kinds of explants including leaf-discs, stem and petiole segments were chosen to evaluate their differentiation capacity. As shown in Fig 1, all the explants generated calli within 30 days. The calli formed from petiole segments (Fig 1A) and leaf-discs (Fig 1C) were a cream yellow color and friable in structure. The calli derived from petiole segments were bigger than from leaf-discs after 30 days of cultivation. In contrast, the calli formed from stem segments were white with dehydrated structure (Fig 1B). Therefore, petiole segments were selected as suitable explants for the following transformation.

### Optimal kanamycin concentration of callus formation

**Fig 2:**
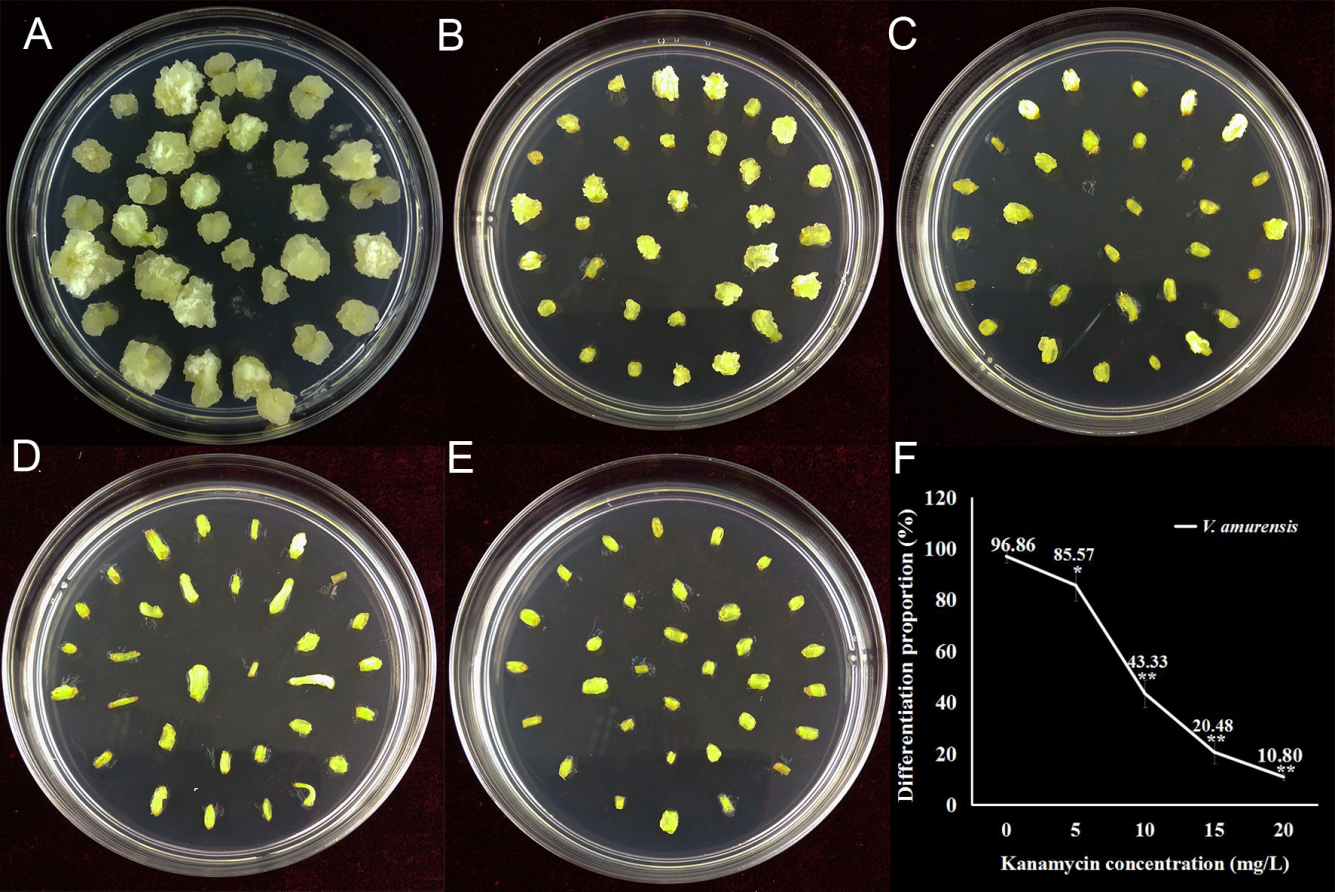
The effect of kanamycin on callus formation from petiole segments. A: Calli generated from petiole segments on medium without kanamycin for 30 days; B-E: Petiole segments on medium containing 5, 10, 15 and 20 mg/L kanamycin, respectively; F: The calculated differentiation proportion of petiole segments under different kanamycin concentrations. Data are the mean values ± SE of three biological replicates. * and ** indicate significant differences between control and treatments at *P*<0.05 or *P*<0.01 (Student’s t-test), respectively.

Kanamycin is a widely used antibiotic in grape post-transformation selection, and the tolerance of different tissues to kanamycin is quite variable [10, 16, 36, 37]. In order to find a suitable concentration of kanamycin for transgenic calli selection, petiole segments were cultured on Petri dishes with medium containing 5, 10, 15 or 20 mg L^-1^ kanamycin for 30 days in the dark at 26°C. Petiole segments that were on similar medium without kanamycin were used as control. The differentiation proportion, defined as the percentage of petiole segments that showed visible callus, was used in evaluating the inhibition of kanamycin during callus formation. As shown in Fig 2A, almost all of the petiole segments (96.86%) produced callus without kanamycin. Conversely, when kanamycin was added to the media, the differentiation proportion declined rapidly, showing a negative correlation with the concentration of kanamycin (Fig 2B–2E and 2F). When 15 and 20 mg L^-1^ kanamycin were added to the media, only 20.48% and 10.8% of petiole segments produced callus, respectively. It was noted that the growth of the calli was also severely inhibited when the concentration of kanamycin was increased. After 30 days culture, calli that were less than 1 mm^3^ were found on petiole segments when 15 mg L^-1^ kanamycin was added to the medium (Fig 2D), while nearly invisible calli were found on medium with 20 mg L^-1^ kanamycin (Fig 2E), which indicated that the differentiation and proliferation of nontransformed calli from petiole segments were very inhibited. Thus, 20 mg L^-1^ kanamycin was employed for selecting positive transgenic calli from petiole segments.

### *Agrobacterium* strain

**Fig 3:**
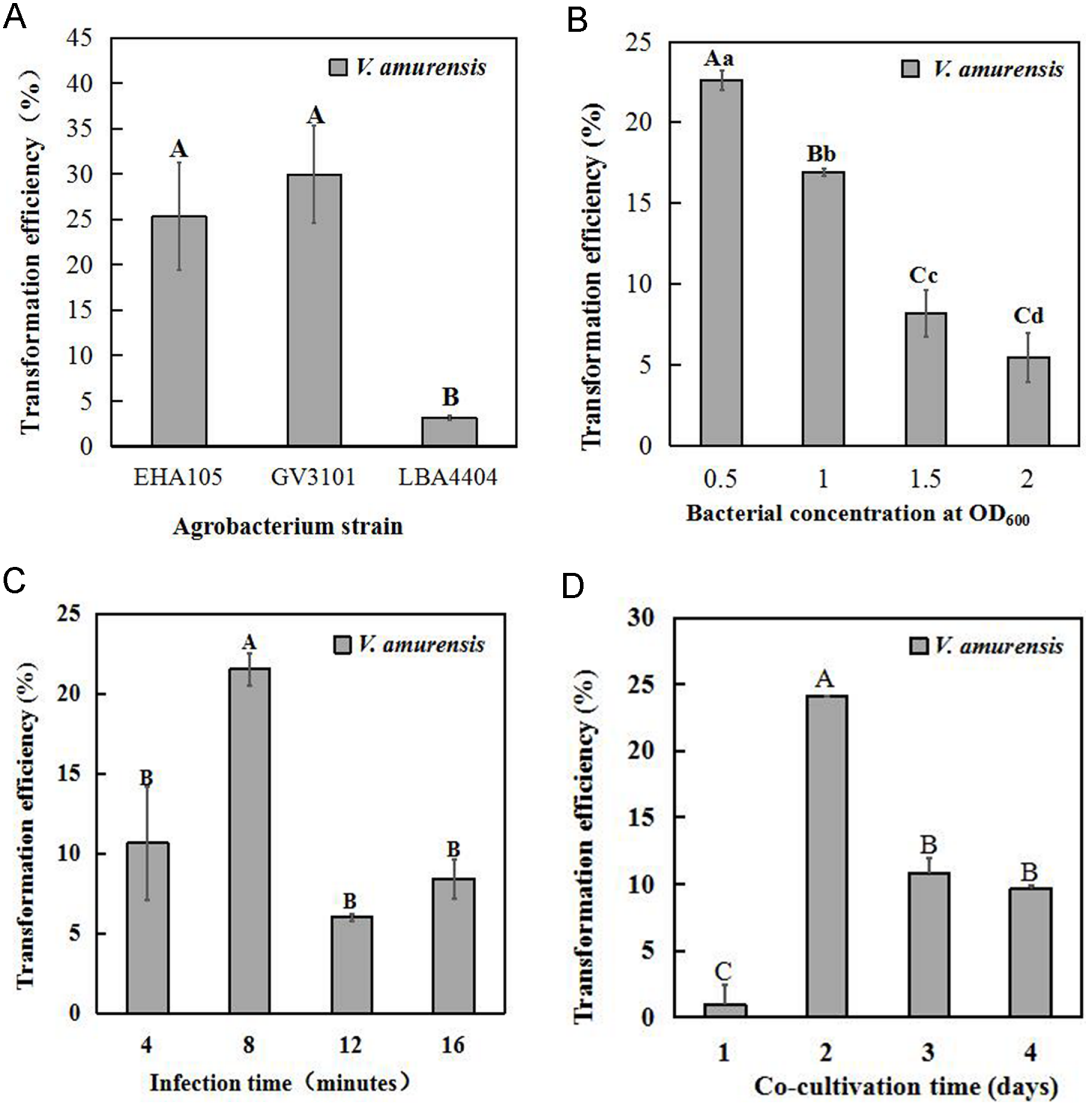
Optimizing parameters for *Agrobacterium*-mediated petiole segment transformation in *V*. *amurensis*. A: The transformation efficiency of petiole segments on different *Agrobacterium* strains (EHA105, GV3101 and LBA4404). B: The effect of bacterial concentration (OD_600_ = 0.5, 1.0, 1.5 and 2.0) during transformation. C: The effect of infection time (4, 8, 12 and 16 min) on transformation. D: The effect of co-cultivation times (1, 2, 3 and 4 days) on transformation. All of the data are the mean values ± SE of three biological replicates. Lower and upper case letters indicate significant differences between treatments at *P*<0.05 or *P*<0.01 (Student’s t-test), respectively.

Three *Agrobacterium* strains (EHA105, LBA4404 and GV3101) are commonly used for *Agrobacterium*-mediated transformation in plants. Here, these strains were also used to assess their transformation efficiency in a petiole segment-based transformation system. A binary vector, pSAK277-eGFP, was transferred into all three strains. After re-cultivation, the suspended bacteria (OD_600_ = 0.5) were used to infect petiole segments. The eGFP fluorescence was detected by microscope to confirm successful transformation in calli. As shown in Fig 3A, the highest transformation efficiency (29.91%) was achieved by using GV3101. In comparison with GV3101, EHA105 had a similar transformation efficiency (25.31%) and no statistically significant difference was found between these two strains. In contrast, only 3.14% of petiole segments produced positive calli during LBA4404-mediated transformation. These results indicated that both GV3101 and EHA105 were suitable for petiole segment transformation in *V*. *amurensis*.

### Optimized procedure for *Agrobacterium*-mediated petiole segment transformation in *V*. *amurensis*

During *Agrobacterium*-mediated transformation in plants, the density of the bacteria, infection and co-cultivation time are the key factors that affect transformation efficiency. Here, the *A*. *tumefaciens* strain EHA105, containing a binary vector pSAK277-eGFP, was used in the following experiment to optimize the parameters during petiole segment transformation.

#### Bacterial density

In order to discover the effect of bacterial density during transformation, re-cultivated cells at different concentrations (OD_600_ = 0.5, 1.0, 1.5 and 2.0) were used to infect the petiole segments. After 8 min, petiole segments were co-cultivated with bacteria on solid medium for 2 days. Petiole segments with transgenic callus were counted after two months and, as shown in Fig 3B, transformation efficiency was up to 22.6% when using re-cultivated bacteria that had an OD_600_ at 0.5. Acceptable transformation efficiency (16.9%) was also achieved from the bacteria that had an OD_600_ at 1.0. The percentage of petiole segments which formed positive callus declined when the density of bacteria was increased during infection. It was observed that only 5.48% of the segments could produce transgenic callus when OD_600_ was at 2.0 for re-cultivated bacteria, which indicated that a high concentration of bacteria inhibited the *Agrobacterium*- mediated transformation process.

#### Infection time

Based on the results from bacterial density analysis, the evaluation of a suitable infection time with bacteria that had an OD_600_ at 0.5 during the transformation processes was conducted. Four time spans (4, 8, 12 and 16 min) were chosen for infection according to previous reports [19, 37], and two days co-cultivation was performed. As the results show in Fig 3C, transformation efficiency varied greatly when different infection times were used. The highest transformation efficiency (21.51%) was achieved when petiole segments were infected for 8 min. When shorter or longer infection times were used, the transformation efficiency dropped dramatically. These results imply that 8 min is the best infection time for petiole segment transformation, coupled with bacterial density (OD_600_ = 0.5) and 2 days co-cultivation.

#### Co-cultivation

Co-cultivation treatment is a post-transformation step which is used to increase transformation efficiency [19, 38]. An inappropriate co-cultivation treatment may reduce the yields of transgenic calli and also increase the risk of unmanageable *Agrobacteriu*m contamination during positive calli selection. So, after infection (8 min), four co-cultivation treatments (the same medium but with different periods of time including 1, 2, 3 and 4 days) were tested. As shown in Fig 3D, the transformation efficiency of 1 day of co-cultivation treatment was only 1.01%, which indicated an insufficient period for the interactions between petiole segments and bacteria. The highest transformation efficiency (24.14%) occurred with 2 days co-cultivation. Further, longer co-cultivation treatments of more than 2 days decreased transformation efficiency (10.83% for 3 days, 9.69% for 4 days). The lower transformation efficiency may be due to the browning at the end of some petiole segments, where the transgenic calli may originate after 3 or 4 days co-cultivation (data not shown). Thus,the 2 days co-cultivation treatment was the best period of time to achieve more transgenic callus lines.

### The eGFP fluorescence observation and molecular analysis of transformants

**Fig 4:**
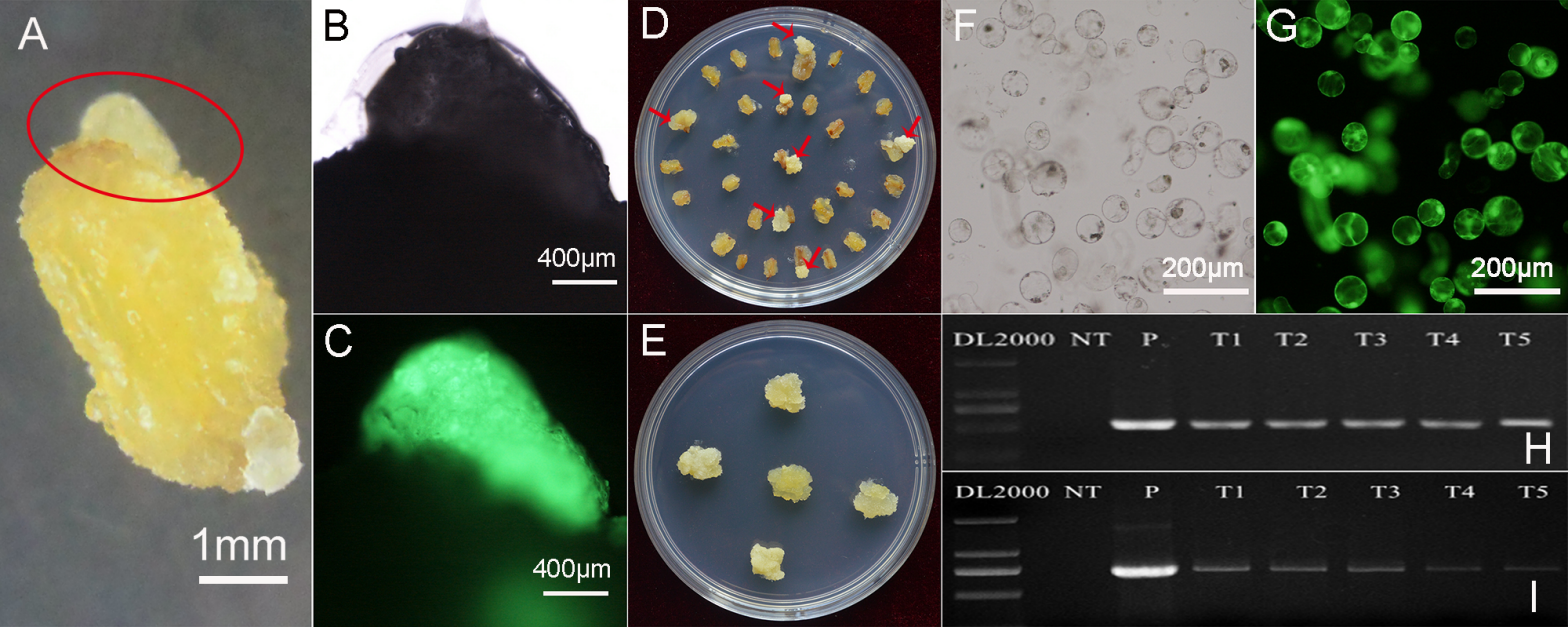
eGFP fluorescence observation and molecular analysis of transformants. A: Transformed calli generated from the end of the petiole segments of *V*. *amurensis* after 30 days cultivation. B, C: Bright-field and eGFP fluorescence results of callus emphasized by a red mark in A. D: Transformed calli generated from the end of the petiole segments of *V*. *amurensis* after 60 days cultivation. E: Different transgenic callus lines were cultivated in new medium. F, G: Bright-field and eGFP fluorescence results of digested cells from one transgenic callus line. H: Amplification of *NPTII* gene fragments from the genomic DNAs of non-transformed calli (NT), pSAK277-eGFP plasmid (P) and 5 transgenic lines (T1-T5). I: Amplification of *eGFP* gene fragments from the cDNAs of non-transformed calli (NT) and 5 transgenic lines (T1-T5). The plasmid of pSAK277-eGFP (P) was used as positive control for PCR amplification. Fluorescence microscope (ECLIPSE 80i, Nikon). GFPuv was excited at 465–495 nm and emitted through a 515–555 nm bandpass filter.

The optimized parameters above were used for *V*. *amurensis* petiole segment transformation. Almost all calli were generated from the ends of the segments on kanamycin-containing medium after one month cultivation (Fig 4A). The callus was pale yellow and easy to distinguish from the segments. The expression of *eGFP* gene was confirmed by strong eGFP fluorescence observed from newly-formed calli (Fig 4B and 4C), which indicated that 20 mg L^-1^ kanamycin was sufficient to inhibit the formation of un-transformed calli. The petiole segments were transferred into new medium monthly until the callus was big enough to cultivate separately (Fig 4D). The different transgenic callus lines (Fig 4E) were divided into small patches respectively, for proliferation on new medium. About 3 g of callus were produced after one month cultivation for each transgenic callus line, which provided enough material for transcription, translation and metabolism-related analyses.

The possible chimera of transgenic calli with un-transformed cells was analyzed by detecting the eGFP fluorescence in detached cells of calli. A total of five transgenic callus lines were detected, and cells were collected by digesting the calli with cellulase and macerozyme. The bright-field and fluorescence detection results of one transgenic callus line is shown in Fig 4F and 4G. The green fluorescence of eGFP was detected in all of the cells from five transgenic lines. The eGFP fluorescence was found both in the nucleus and cytoplasm, which was in consensus with its localization in other plant cells. These results demonstrated that no un-transformed cells were interfused into transgenic callus.

The DNA and total RNA were isolated from non-transformed calli and five transgenic callus lines where the insertion of the *NPTII* gene into the callus genome and the transcription of *eGFP* gene were checked. A 515 bp *NPTII* gene fragment was successfully amplified from pSAK277-eGFP plasmid and all transgenic callus lines (Fig 4H) but failed from non-transformed calli. The expression analysis of *eGPF* gene also showed similar results (Fig 4I). These results, combined with observed fluorescence from eGFP, confirmed the successful transformation of target genes into the calli from *V*. *amurensis* petiole segments.

## Discussion

It was reported that transformation in grapevines is dramatically affected by the species, cultivar and explant tissues used [14, 20, 39]. Here, a petiole segment-based transformation system was established for *V*. *amurensis*. However when two *V*. *vinifera* cultivars (‘Muscat Hamburg’ and ‘Centennial Seedless’) were used with the optimized procedure used for *V*. *amurensis* petiole segments, almost negligible transgenic callus (a transformation efficiency less than 1%) was generated at the ends of petiole segments. This result implied that genetic background is key for successful petiole segment transformations.

Agroinfiltration-based transient transformation systems were established using grapevine leaves, and stable transformed cell lines were generated in *V*. *vinifera* cv. Sugraone [40]. Here, petiole segments from plantlets were chosen as explants due to their excellent differentiation capacity. Sterile plantlets of *V*. *amurensis* were cultivated in conical flasks and proliferated for 6 weeks, which facilitated petiole collection without any restrictions. *V*. *amurensis* petioles from the vineyard were also used at the beginning of explant selection. Petioles were cut after sterilization with 70% alcohol and 2% hypochlorite. When petioles from the vineyard were used, necrotic tissue was evident at the end of the petiole segments and callus was not formed after 2 months, which indicated that either the sterilization condition or concentration of kanamycin were not suitable for vineyard–derived petioles.

Hygromycin and kanamycin are used as selection markers for grapevine transformation [10, 19]. In comparison with kanamycin, explants from grapevine were more sensitive to hygromycin used at 2–10 mg L^-1^ for selection [19, 41]. In our pre-experiment, 10 mg L^-1^ hygromycin completely inhibited callus generation from petiole segments. The appropriate selection concentration of hygromycin was difficult to ascertain in petiole segment cultivation. Therefore, pSAK277 vector which contained *NPTII* gene was chosen and kanamycin was used as the selectable marker in petiole segment transformation. The concentration of kanamycin used for selection varied from 15–300 mg L^-1^ in somatic embryogenesis-based transformation [16, 17, 18, 19, 42]. In this study, an optimal concentration of kanamycin at 20 mg L^-1^ was chosen and confirmed by eGFP fluorescence observation. No chimeric cells were found in selected calli which also supported use of the concentration of kanamycin chosen in petiole segment transformation for transgenic callus selection.

The effect of *A*. *tumefaciens* strain on grapevine transformation has been previously described [16, 40]. Torregrosa *et al*. found that super-virulent strain EHA105 increased transformation efficiency when compared to the widely used strain LBA4404[14]. This observation is in accordance with our results since strain EHA105 showed higher transformation efficiency than strain LBA4404. In addition, the strain of GV3101 has a similar performance and both it and EHA105 were usable in petiole segment transformation. Besides the strains, the density of bacteria, infection treatment and co-cultivation time affected the transformation process. The high concentration of bacteria and long infection treatment and co-cultivation periods prohibited the formation of transgenic calli from petiole segments (Fig 3). Ahmed *et al*. also reported that the transformation efficiency of embryos dropped when inoculation of more than 10 min or co-cultivation with bacteria for more than 2 days was used [19]. This may explain the host/pathogen interaction between *Agrobacterium* and grape cells which leads to cell death and tissue necrosis [43]. Thus, in our experiment, a low concentration of bacteria (OD_600_ = 0.5) with moderate infection and co-cultivation periods of 8 min and 2 days, respectively, were used to increase transformation efficiency.

Here, we established a quick and efficient system to obtain transformed calli of *V*. *amurensis*. In one transformation, more than 20 transgenic callus lines were obtained simultaneously during transformation using 100 petiole segments. Generation of sufficient materials from different transgenic callus lines were achieved within 3 months, which could be used for transcription, translation and metabolism-related studies in grapevines, especially in *V*. *amurensis*. This transformation system will be vital in studying the function of cold stress-related genes, where such transcription factors will accelerate the explanation of cold acclimation mechanisms in *V*. *amurensis*. In recent years, a large numbers of genes related to fruit quality and stress responses in grapevines have been widely identified courtesy of published genome sequences and powerful omics research platforms [44–46]. This petiole segment transformation system also provides an ideal assay platform for quickly identifying key genes in these biological processes.

## Supporting information

S1 Fig The diagram of pSAK277-eGFP vector construction. (DOCX)

## Abbreviations

2,4-D: 2,4-Dichlorophenoxyacetic acid
6-BA: 6-Benzyladenine
AS: Acetosyringone
BSA: Bovine serum albumin
eGFP: Enhanced green fluorescent protein
IAA: Indole-3-acetic acid
MES: 4-morpholinoethanesulphonic acid
MS: Murashige and Skoog’s medium
NPTII: Neomycin phosphotransferase
PVP: Polyvinyl pyrrolidone
