# Supplementary figures and images for: An efficient method for transgenic callus induction from Vitis amurensis petiole
Source: PLoS One. 2017 Jun 22;12(6):e0179730. doi: 10.1371/journal.pone.0179730 (PMC5481001; doi:10.1371/journal.pone.0179730)

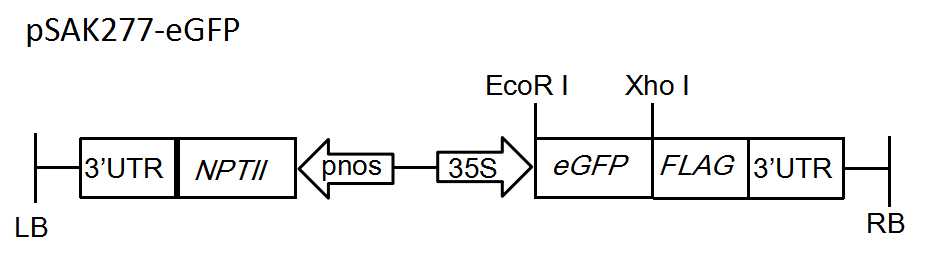


S1 Fig. The diagram of pSAK277-eGFP vector construction.

Supplement: S1 Fig — (DOCX) [file pone.0179730.s001.docx]
